# Large-scale microsatellite development in grasspea (*Lathyrus sativus* L.), an orphan legume of the arid areas

**DOI:** 10.1186/1471-2229-14-65

**Published:** 2014-03-17

**Authors:** Tao Yang, Junye Jiang, Marina Burlyaeva, Jinguo Hu, Clarice J Coyne, Shiv Kumar, Robert Redden, Xuelian Sun, Fang Wang, Jianwu Chang, Xiaopeng Hao, Jianping Guan, Xuxiao Zong

**Affiliations:** 1 The National Key Facility for Crop Gene Resources and Genetic Improvement/Institute of Crop Science, Chinese Academy of Agricultural Sciences, Beijing 100081, China; 2 Department of Leguminous Crops Genetic Resources, N. I. Vavilov Research Institute of Plant Industry, St. Petersburg 190000, Russia; 3 USDA-ARS Western Regional Plant Introduction Station (WRPIS), Pullman, WA 99164, USA; 4 International Center for Agricultural Research in the Dry Areas (ICARDA), Aleppo 5466, Syria; 5 Australian Temperate Field Crops Collection, Grains Innovation Park, The Department of Primary Industries, Private Bag 260, Horsham, Victoria 3401, Australia; 6 The Key Laboratory of Crop Gene Resources and Germplasm Enhancement on Loess Plateau of Ministry of Agriculture/Institute of Crop Germplasm Resources, Shanxi Academy of Agricultural Sciences, Taiyuan 030031, China

**Keywords:** *Lathyrus sativus* L, Microsatellite, 454 FLX Titanium pyrosequencing, Marker development

## Abstract

**Background:**

Grasspea (*Lathyrus sativus* L., 2n = 14), a member of the family Leguminosae, holds great agronomic potential as grain and forage legume crop in the arid areas for its superb resilience to abiotic stresses such as drought, flood and salinity. The crop could not make much progress through conventional breeding in the past, and there are hardly any detailed molecular biology studies due to paucity of reliable molecular markers representative of the entire genome.

**Results:**

Using the 454 FLX Titanium pyrosequencing technique, 651,827 simple sequence repeat (SSR) loci were identified and 50,144 nonredundant primer pairs were successfully designed, of which 288 were randomly selected for validation among 23 *L. sativus* and one *L. cicera* accessions of diverse provenance. 74 were polymorphic, 70 monomorphic, and 144 with no PCR product. The number of observed alleles ranged from two to five, the observed heterozygosity from 0 to 0.9545, and Shannon’s information index ranged from 0.1013 to 1.0980, respectively. The dendrogram constructed by using unweighted pair group method with arithmetic mean (UPGMA) based on Nei's genetic distance, showed obvious distinctions and understandable relationships among the 24 accessions.

**Conclusions:**

The large number of SSR primer pairs developed in this study would make a significant contribution to genomics enabled improvement of grasspea.

## Background

Grasspea (*Lathyrus sativus* L.) is an excellent candidate crop to provide protein and starch for human diets and animal feeds in the arid areas [1]. It is one of the hardiest crops for adaptation to climate change because of its ability to survive drought, flood and salinity [2]. It also plays a vital role in many low input farming systems [3]. However, undesirable features such as prostrate plant habit, indeterminate growth, pod shattering, later maturity and presence of neurotoxin, β-N-oxalyl-L-α,β-diaminopropionic acid (β-ODAP), limit its cultivation under various agro-ecological conditions [4-6].

To date, less than 205 microsatellite (SSR) markers have been published for grasspea, and only 61 of them were characterized for size polymorphism [7-9]. Lioi et al., [7] searched for the presence of SSRs with the European Molecular Biology Laboratory (EMBL) nucleotide sequence database. Ten out of 20 SSR primers were successfully amplified, and only six of them exhibited size polymorphism. In addition, Ponnaiah et al., [8] searched for EST-SSRs in the National Center for Biotechnology Information (NCBI) database. Seven of the 19 *Lathyrus* EST-SSRs and four of the 24 *Medicago* EST-SSRs revealed polymorphism when screening *L. sativus* accessions [8]. Sun et al., [9] analyzed a total of 8,880 *Lathyrus* genus ESTs from the NCBI database (up to March 2011), identified 300 EST–SSR and designed primers to characterize for size polymorphism among 24 grasspea accessions. Among them 44 SSR markers were polymorphic, 117 markers monomorphic and 139 markers with no bands [9]. Lioi sequenced 400 randomly selected clones and get 119 retrieving SSR containing sequences. 7 primer pairs produced clearly distinguishable DNA banding patterns in 10 randomly selected SSRs, The transferability of SSR markers was high among three related species of *Lathyrus*, namely *Lathyrus cicera*, *Lathyrus ochrus* and *Lathyrus tingitanus*, and the legume crop, *Pisum sativum*[10].

Next generation sequencing (NGS) technologies has become popular on its success of sequencing DNA at unprecedented speed thereby enabling impressive scientific achievements and novel biological applications [11,12]. Next generation RNA sequencing (RNA-Seq) is rapidly replacing microarrays as the technology of choice for whole-transcriptome studies [13]. RNA-Seq also provides a far more precise measurement of levels of transcripts and their isoforms than other methods [14]. However, few studies solely focused on high-throughput novel microsatellite markers discovery of orphan crops via next generation sequencing [15-19].

Recently, we applied next generation sequencing to obtain high-quality putative SSR loci and flanking primer sequences inexpensively and efficiently. The novel SSR sequences were characterized and validated through successful amplification of randomly selected primer pairs across a selection of 23 grasspea accessions and one accession of its direct ancestor red pea (*Lathyrus cicera*) as an outgroup.

## Methods

### Plant material

Eight grasspea (*L. sativus*) accessions consisted of two Chinese, two Asian, one African and three European accessions were used for the 454 sequencing.

A set of 23 grasspea (*L. sativus*) accessions and one red pea (*L. cicera*) accession were used in SSR marker testing and genetic diversity analysis. These genetic resources contained six accessions from China, seven each from Asia (including one *L. cicera* accession) and Europe, and four from Africa.

The seed samples were obtained from the National Genebank of China at Institute of Crop Science, Chinese Academy of Agricultural Sciences, Beijing, China. Details information is given in Additional file 1: Table S1.

### DNA isolation, library preparation and 454 sequencing

The sprouts from each of the eight genotypes were collected and total genomic DNA was isolated using the CTAB method from the seven-day old seedlings grown under dark condition at 18°C. A selective hybridization with streptavidin coated bead method was used to construct SSR-enriched genomic libraries. The following eight probes were used: p(AC)_10_, p(GA)_10_, p(AAC)_8_, p(AAG)_8_, p(AAT)_8_, p(ATGT)_6_, p(GATA)_6_ and p(AAAT)_6_. Libraries quality control was conducted by randomly selecting and sequencing 186 clones. The DNA fragments were inserted into pGEM-T EASY vector, and insert fragments were validated by Sanger sequencing. If the libraries had high ratio of insert fragments and most fragments length were from 500 to 800, they were considered as high quality.

The eight SSR-enriched DNA libraries were equally pooled for pyrosequencing using the 454 Genome Sequencer FLX Titanium System at Beijing Autolab Biotechnology Co. Ltd (China). Finally, the 454 System collected the data and generated standard flow gram file (.sff) which contained raw data for all the reads. Then, grasspea.sff file was submitted to the sequence read archive (SRA) at the National Center for Biotechnology Information (NCBI) with the accession number SRX272771.

### Reads characterization

All high quality reads were processed to remove adaptor-ligated regions using the Vectorstrip program in EMBOSS software package [20]. Moreover, in-house developed program such as: SeqTools.pl, ACGT.pl, ave_length.pl, and max.pl programs were used to analyze the total number of nucleotide A, T, C, G in all reads, the average length of all read sequences, and the maximum length read in our study.

### SSRs searching

Before SSRs searching, “clean reads” were filtered redundant at 98% sequence identity, using CD-HIT program (http://weizhong-lab.ucsd.edu/cd-hit/). A high-throughput SSR search was performed using MISA (Microsatellite identification) tool (http://pgrc.ipk-gatersleben.de/misa/). The parameters were as following: minimum SSR motif length of 10 bp and repeat length of mono-10, di-6, tri-5, tetra-5, penta-5, and hexa-5. The maximum size of interruption allowed between two different SSR in a compound sequence was 100 bp.

### SSR characterization

The MISA file was used to analyse the number of sequences containing SSRs, the number of SSRs detected, the number of SSRs starting within 200 bp of read sequences, the dominant types of SSR motifs within mono-, di-, tri-, tetra-, penta- and hexa- repeats, and the ratio of single, perfect compound and interrupted compound SSRs. These characterizations were obtained by statistical analysis from the MISA files [21] by a small Perl program and plotted by R language [22], and OpenOffice.org Calc.

### Primer pairs designing

Primer pairs were designed by Primer 3.0 interface modules containing p3_in.pl Primer 3.0 [23] and p3_out.pl files (http://pgrc.ipk-gatersleben.de/misa/primer3.html). These Perl scripts were used to normalize the format in order to design primers flanking the microsatellite locus. Amplification product sizes ranged from 100 to 300 bp. Then, the in-house developed script primer_random_pick.pl was used to gain the non-redundant primers.

### Polymerase chain reactions (PCR) amplification

For each of primer pair, PCRs were performed twice, each time with a different Taq enzyme and reaction buffer. All the primer pairs were amplified in the first round experiment with 20 μl reaction volumes containing 0.5 U of TaKaRa Taq polymerase (Code No.: R001A, TaKaRa, Dalian, China), 2 μl of 10 × PCR Buffer (Mg^2+^ plus), 0.2 μl of dNTP (2.5 mM each), 0.4 μM primer, and 50 ng of genomic DNA. Then the no bands or weak bands primers were used in the second round PCR reaction using TAKaRa LA Taq polymerase with GC buffer (Code No.: RR02AG, TaKaRa, Dalian, China) according to the manufacturer’s instructions. SSRs were amplified on Heijingang Thermal Cycler (Eastwin, Beijing, China). Under the following conditions: 5 min initial denaturation at 95°C; 35 cycles of 30 s at 95°C, 30 s at the optimized annealing temperature (Table 1), 45 s of elongation at 72°C, and a final extension at 72°C for 10 min. PCR products were tested for polymorphism using 6% denaturing polyacrylamide gels and visualized by silver nitrate staining.

**Table 1:** Characteristics of 74 polymorphic microsatellite loci developed in grasspea (FP = forward primer, RP = reverse primer, Ta = annealing temperature)

**Primer**	**Repeat motif**	**Primer sequence (5′-3′)**	**Real product size(bp)**	**Ta/°C**
G1	(A)10	FP-AAGGAGCAGCAGCATTTGTT	210-240	52
		RP-TAATAATGGGGAGCCGATCA		
G4	(AAAG)5	FP- CCTTTCGGAGCAATCAAGAC	110-120	56
		RP- TGCCTAAGCATTGGCTTTCT		
G5	(AAC)10	FP- CACAACCAGTTGCATCAGTG	200-220	56
		RP- TGGCTCACATGATGGTTTGT		
G6	(AAC)12	FP- TGGAGGACGAGCAACAATAA	230-250	54
		RP- TGTTGTTGATGGAAACAAATGA		
G7	(AAC)5	FP- ACAGCAAGAAGCAGCAACAG	230-245	56
		RP- AGTTGGTTGTTGTGTCGTTGT		
G9	(AAC)6	FP- CAACCAGAGCAACCACAAGA	240-260	56
		RP- GGTTGCAAGAGGTTGCAGAT		
G13	(AACCA)5	FP-CAAACCAAACCAAACCAAACT	180-195	52
		RP- CGCGTTTTGGTTTTCGTACT		
G15	(AAG)6	FP- TCAAGCCCAAAGTGAGATGA	145-155	56
		RP- TTTTGTGTTGCTTGCTGACC		
G17	(AAT)5	FP- CAGGTCCGGCTTATCTCTCA	180-200	56
		RP- TTGGTTTCAACCCACTCCTC		
G18	(AC)10	FP- ACACGACACACACGACAGTG	130-140	52
		RP- CTGCGTGTCTGTGCCTATTG		
G27	(AC)18	FP- ATCTTACCGGGGATCCATTC	190-210	56
		RP- CTTCCCCATTCTCTGGTGTT		
G33	(AC)6	FP- ACCAAAGGATGCAGGGTCTA	230-270	54
		RP- TAGTCGTGGTGTCGTGGTGT		
G39	(AC)6	FP- CCAGACACACACGCAAACAT	170-190	54
		RP- GTGTGTGACGTTGCCGTTAG		
G49	(AC)7	FP-ACGCACACACGGAAGAAAG	160-180	56
		RP-GTGTGCGCATGTGTGTATGA		
G61	(AC)8	FP-CACACACCATTACGCACACA	130-150	54
		RP-TGGTGTCGTGGTCGTAGGTA		
G64	(AC)8	FP-GCACATTCGCACGTATTCAC	160-180	56
		RP-CGTTTCTGAGTGCGTTGTGT		
G67	(AC)9	FP-CACCCTCTTCACTGCCTAGC	120-150	52
		RP-TTGGGGGTTGTAGAAGGAAC		
G68	(AC)9	FP-GCACACAAGGGCACACTG	170-180	52
		RP-TGCGTCGTGTGTATGTGTTG		
G72	(ACA)5	FP-CAACGACAACAACGCAAAAC	260-280	52
		RP-TTCGCGGTTTGTCCATTTAG		
G73	(ACA)5	FP-CCAACTCTCAGCCACGAACT	200-220	54
		RP-TTGCTCCACCTACGCTTCTT		
G75	(ACA)6	FP-AACAACAGCAGCAACAACAAT	200-215	54
		RP-CGTGTTGTGTGTTCGTTCGTA		
G76	(ACA)6	FP-CACAACCAACGCCAATACAG	230-250	54
		RP-CCGTAGTACCGCGCTTATTC		
G77	(AAC)11	FP-ACAAGACAACATCACCGAGAC	300-330	52
		RP-TGTTGTTTGGTTGTTCGTGTA		
G80	(AAC)5	FP-AAACACAACAGACGATTAAACACA	185-200	52
		RP-TCTTGCTATGTAGTGTTGTGTGATG		
G81	(ACG)5	FP-CGCACACACTCACACACAAC	180-200	52
		RP-GGTCCTGTCGTCGTAGTCCT		
G83	(ACG)7	FP-GGGCACACATTCTCACACAC	190-200	54
		RP-TGTCGTCGTGTCGTAGTCGT		
G87	(AG)15	FP-CCCTTACCGAGTGCAGAAAA	230-250	54
		RP-CACCACGACTTGCTCACCTA		
G101	(CA)11c(CA)7	FP-TGGCAGGTAACTGGTGAGTG	180-190	52
		RP-GGTGTTTCCCCACCTCTCTA		
G102	(CA)12	FP-AAAGCACAGCACAACACGAC	260-280	54
		RP-AACAAGGACGACGGTAGGTG		
G110	(CA)6	FP-CACAAACACGCACAAACACA	150-170	52
		RP-CGTCGGTATAACCGTGTCGT		
G116	(CA)6(CACACG)5	FP-CACACAGGACAGCACTCACA	150-180	52
		RP-GTCGTCGGTGTGTCGTAGTC		
G119	(CA)6cgacacacncgcgcgcgcgacacac(ACG)8	FP-CGTCTCTTCAAAGGGCCATA	190-200	52
		RP-CGACCGACCGACGTACTACT		
G120	(CA)6cgcacgcacgcacacagacacg(CA)7	FP-GCGCACGCATACATACACA	160-170	54
		RP-TTGCCGTTGTCGTGTTAGTG		
G123	(CA)6gn(AC)6	FP-CATAACAACACGCAGCATTACC	130-140	52
		RP-TTGCGTTGTTGTGTTGTGTTT		
G128	(CA)7	FP-CCACACACCCACATGTTCA	210-230	56
		RP-TTGTGGTGGGTCTGAGAGTG		
G131	(CA)7aacacgttcg(CA)8	FP-GCGCTCACACCAACATAAAG	140-150	54
		RP-TGTATGCGTGCGTATGTCTG		
G133	(CA)7cgcacat(AC)6	FP-ACGCGTGCACACATTTTATC	200-220	52
		RP-TATGTGGGCGCGTGTAAGTA		
G136	(CA)7tacacacacat(AC)7aa(AC)6	FP-ACGACGACCACCAGTACGA	110-130	54
		RP-ACGAGTGCGTGTGTGAGTGT		
G142	(CA)8cgcacaa(AC)10	FP-CGTGCACGCACAGATACG	160-180	52
		RP-GTGTGTGTGTTCGTCGTTTG		
G143	(CA)8cggcgcgcg(AC)9	FP-GACACACACAACCCGAACAC	230-260	52
		RP-TGAGCGAACGTACGTGGTAG		
G145	(CA)8tacgcacg(CA)10	FP-ATACAAGCACGCATCCACAG	100-120	52
		RP-AGTTCGTGTCGTGTCGTGTC		
G147	(CA)9	FP-CGTCACACACGTCACGTACA	210-230	54
		RP-CTACGAGACGCACGACGATA		
G150	(CA)9 g(AC)25	FP-CACACACACCAAGCGTTACA	140-160	54
		RP-TCGTGTGTGTCGTGTGTGTAG		
G151	(CAA)10	FP-CAACAACGACAACAAAATTGTAA	175-185	52
		RP-CTGCTGATGTTGTTGGTGCT		
G154	(CAA)5agaccacaacaccaccacc aacaacaacaataataaaacag(AAC)5	FP-CTGGCGTAATAGCGAAGAGG	240-260	56
		RP-TGTGTTGCTTTGTGTTGTCGT		
G157	(CAA)6	FP-ACATCCAATCCCCACCATAA	210-230	56
		RP-AATGCATGGTTGTTGCTTGA		
G165	(CGA)5	FP-GAACGTACGACGACACGAACT	270-290	54
		RP-CGTGTGGTGTGTGTGTGTGT		
G171	(CT)9	FP-CTTCACTGCATGCTTTCCAC	200-230	52
		RP-CTGGGGTGGTTTTTGTCAGT		
G174	(GA)19	FP-CACAAGGGTCAAGGGAGAGA	140-160	52
		RP-GTTTACGTTACTTATTCGTTCGTTAG		
G184	(GT)15	FP-GCGTGTGTGCGAATGTGT	180-190	52
		RP-CACGCACGCACACTAGACTAC		
G185	(GT)19	FP-TGCGTGTGTCGCTCTATCAT	130-135	56
		RP-TACTGCGACAACCGAACGTA		
G188	(GT)6	FP-GCGCGTTAGTGTGTGTTTGA	140-150	52
		RP-CACGCACGCACACTTACATA		
G191	(GT)6	FP-TGTGCGTGGTGTTTGAGTG	140-160	52
		RP-CACATACGCACAGCCCATAC		
G192	(GT)6a(TG)7	FP-TGCGTGATAAGGTGCTTGAG	160-170	56
		RP-ACACACACACGCACACACAC		
G200	(GT)7	FP-GGATGGTGTGCTGTGTGTGT	120-135	52
		RP-AACACCAACTACCGGCAACT		
G205	(GT)7gcgtgtgcctgcgtctctgcgagtgcgtgc(GT)6	FP-TGTCTGGTGTGTGTGGTGTG	230-250	52
		RP-CGACACGTACGCAACGAC		
G206	(GT)8	FP-AAACTGGCCCTGCATTTTC	190-210	52
		RP-GGTCATGGCAATTTGAGACA		
G209	(GT)8	FP-TTTGCACGTGTCCTGTGTTT	240-260	52
		RP-ACGACGACCACACACCACTA		
G211	(GT)9	FP-ATGGCGTCGTATGTGTGTGT	200-210	52
		RP-GTTACGGCCGAATCAACAAC		
G219	(GTT)10	FP-CCAGTTGTGCCGAACACAT	130-160	52
		RP-CCAACAGCAGATTGCCAGTA		
G225	(GTT)7	FP-GGGCAGTGGACCAGTTAGAG	250-270	52
		RP-CCGAGGGAATAAACGACAAA		
G228	(T)10	FP-CCTACGGACATGCCTGTTTT	280-310	52
		RP-GCGGTAGGGGAAAAACAACT		
G233	(TC)20	FP-CGTTCGTCCTTCTCCTCCTA	120-140	52
		RP-AGACGACTACGGACGACGAC		
G234	(TC)7	FP-GTTGGGTTTGGCATTGAACT	190-210	52
		RP-GAAGGGGCGAACAAATAAAA		
G244	(TG)6	FP-CAATCCGAAAATCACCACCT	230-250	52
		RP-GCACTCACATGCACACAAAC		
G245	(TG)6	FP-CGTTGGTTGTTAGTCGGTCA	240-260	52
		RP-GAACGAAACAACGACGACAA		
G249	(TG)6c(GT)7	FP-TATGTGTGCAACGGCAACTT	140-160	52
		RP-GCACACCCACCACACAATAG		
G254	(TG)7	FP-TGAGTGCGTACGTGTGTCTG	100-120	52
		RP-GCGCGTGTTCACACATAGAC		
G262	(TGGT)5	FP-TGTGCGTGTGTGTGTTTTTG	300-320	52
		RP-ACCACAACCCCTACCCTACC		
G268	(TGT)5tattn(TTG)6	FP-TTGTTTGTTGTTGTTGTGTCTTG	290-305	52
		RP-CTACAGTACAGACCCGCCACT		
G269	(TGT)6	FP-ATGCTGTTGATGCGTCAGTT	220-240	52
		RP-TGCAGCAACAACAAATAAGACA		
G273	(TGT)7	FP- TTTTTGGTATTGTTGTTGTCGT	250-270	52
		RP- CTGCAGCAATAACAGCATCAG		
G284	(TTG)6	FP-TGTGTTGTGTTGTGCTGTATGTA	160-170	52
		RP-GCAGCAACATTAAAACGAACAG		
G285	(TTG)6	FP-TTTGTGCGGTTGATGTTGTT	190-200	52
		RP-CTACGTCAGCCCGTCATACC		

### Evaluation of polymorphic primers in different accessions

288 SSR markers were randomly selected for validation feasibility and size polymorphism among 23 grasspea (*L. sativus*) genotypes from diverse geographical locations and one red pea (*L. cicera*) genotype. POPGEN1.32 [24] software was used to calculate the observed number of alleles (*Na*), the level of observed heterozygosity (*Ho*) and the Shannon’s information index (*I*).

### Genetic diversity analysis

Cluster analysis was conducted based on Nei’s [25] unbiased genetic distance, by using POPGEN1.32 [24] software with the unweighted pair group method on arithmetic averages (UPGMA) algorithm. The resulting clusters were expressed as a dendrogram drawn by MEGA4 [26].

## Results

### Quality control during library construction

The quality of SSR enriched grasspea library was inspected by sequencing 186 randomly selected clones. The resulting data verified that, the recombination rate was 95%, and 29 sequences contained 89 SSR motifs within the cloned sequences.

### 454 sequencing and characterization reads

**Figure 1:**
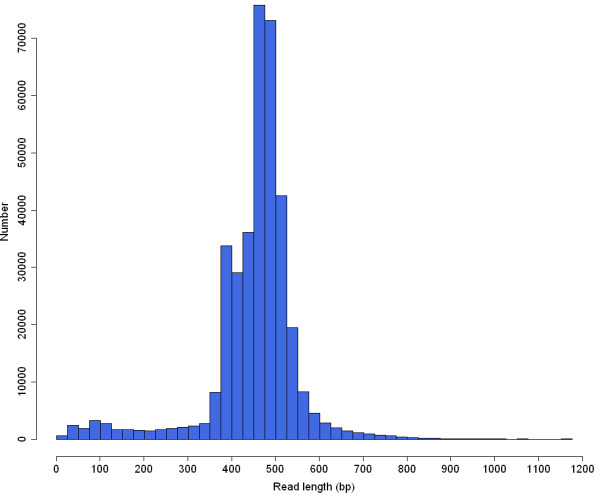
Size distribution of 454 reads.

A total of 493,364 reads were generated from the Roche 454 GS FLX Titanium platform. After adaptor removing, 370,079 read sequences were used for further analysis. The most common nucleotide was thymidine, according for 27.7% of total nucleotides, followed by adenosine (27.2%), guanine (22.2%) and cytosine (22.1%). The mean GC content was 44.3%. The average length of read sequence was 453 bp, with a maximum length of 1,162 bp (Figure 1).

### Mining for SSRs (simple sequence repeats)

**Figure 2:**
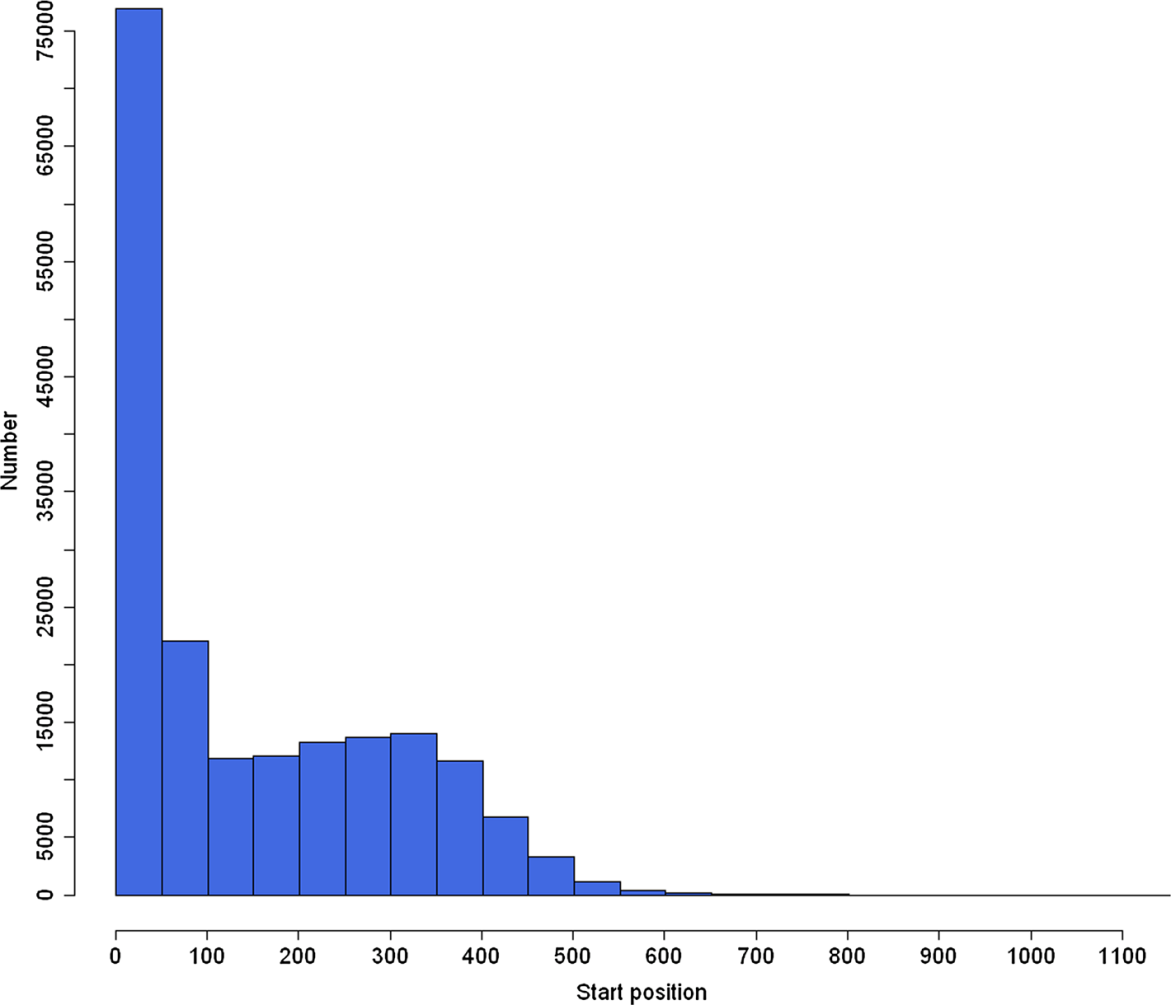
Distribution of SSR motif start position.

Firstly, we employed the program CD-HIT (http://weizhong-lab.ucsd.edu/cd-hit/) to produce a set of 280,791 non-redundant representative sequences. Then, Microsatellite identification tool (MISA) (http://pgrc.ipk-gatersleben.de/misa/) was used for microsatellite mining. As a result, 651,827 SSRs were identified in 129,886 read sequences. Among them, 115,172 read sequences contained more than one SSR. The number of SSRs presenting in compound formation was 464,271 (Table 2), which meant high proportion of SSR loci (71.2%) was located within compound repeats. The majority of identified SSRs (65.4%) were located within 200 bp from the 5′-terminus, and few of SSRs fell into the 3′-terminus (Figure 2).

**Table 2:** MISA result in the genome survey

**Category**	**Numbers**
Total number of sequences examined	280,791
Total size of examined sequences (bp)	130,484,900
Total number of identified SSRs	651,827
Number of SSR containing sequences	129,886
Number of sequences containing more than one SSRs	115,172
Number of SSRs present in compound formation	464,271

### SSR motifs characterizing

**Figure 3:**
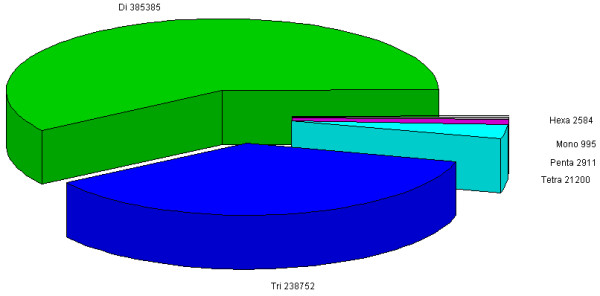
A pie-chart of different SSR motifs in the grasspea sequence data obtained by the current project.

The identified SSRs included 995 (0.2%) mononucleotide repeat motifs, 385,385 (59.1%) dinucleotide repeat motifs, 238,752 (36.6%) trinucleotide repeat motifs, 21,200 (3.3%) tetranucleotide repeat motifs, 2,911 (0.4%) pentanucleotide repeat motifs, and 2,584 (0.4%) hexanucleotide repeat motifs (Figure 3). Thus over 95% of the motifs were di- and tri-nucleotides. The most abundant repeat motif type was (AC/GT)n, followed by (AAC/GTT)n, (AG/CT)n, (ACG/CTG)n and (ACGT/ATGC)n, respectively (Additional file 2: Figure S1, Additional file 3: Figure S2, Additional file 4: Figure S3, Additional file 5: Figure S4, Additional file 6: Figure S5, Additional file 7: FigureS6).

### Compound SSR analysis

In our study, perfect SSRs (i.e., (CA)_8_ which were named as P2 type) were relatively less frequent (29.4%) than the compound SSRs (70.6%). In addition, there were two kinds of compound SSRs: those with interruption between two motifs (i.e., (CT)_8_cacacg(CA)_9_ which were named as C type); and those without interruption between two motifs (i.e., (GT)_6_(GTC)_6_ which were named as C* type). There were 123,444C type (93.2%) and 8,989C* type (6.8%) compound SSRs detected, which suggested the complexity of the grasspea genome.

### Primer pairs designing

A total of 62,342 primer pairs flanking the SSRs were successfully designed using the public shareware Primer 3.0 (http://www-genome.wi.mit.edu/genome_software/other/primer3.html.), based on criteria of melting temperature, GC content and the lack of secondary structure. Furthermore, 50,144 non-redundant primers were achieved by in house developed programs (Additional file 8: Table S2).

### Validation of SSR markers

To validate the SSR sequences, 288 SSR primer pairs were randomly selected for PCR amplification for size polymorphism among 23 grasspea (*L. sativus*) genotypes from diverse geographical locations and one red pea (*L. cicera*) genotype. After two rounds of PCR amplifications, 74 primer pairs were confirmed of being able to amplify polymorphic based across the 24 genotypes (Table 1), 70 primer pairs were confirmed to amplify only monomorphic fragments, and 144 primer pairs produced no products. The number of observed alleles (*Na*) ranged from two to five, the observed heterozygosity (*Ho*) from 0 to 0.9545, and Shannon’s information index (*I*) ranged from 0.1013 to 1.0980 (Table 3). These results indicate the broad utility of the SSR markers obtained from next-generation sequencing for future studies of grasspea genetics.

**Table 3:** **Results of initial primer screening through 24 diversified accessions in *Lathyrus***

**Primer pair ID**			
	***Na***^***1***^	***Ho***^***2***^	***I***^***3***^
G1	3	0.4211	0.8258
G4	2	0.0000	0.1914
G5	2	0.2381	0.5196
G6	2	0.5000	0.5623
G7	2	0.0000	0.1849
G9	3	0.8750	0.7691
G13	2	0.1176	0.5456
G15	2	0.0714	0.1541
G17	2	0.2000	0.3251
G18	4	0.1500	0.5086
G27	4	0.0870	0.7216
G33	3	0.5556	0.7086
G39	3	0.3846	0.7436
G49	5	0.9545	1.0691
G61	4	0.7143	0.9592
G64	3	0.1429	1.0346
G67	5	0.6250	1.0782
G68	2	0.9091	0.6890
G72	2	0.0000	0.2146
G73	3	0.0667	0.7689
G75	2	0.3478	0.4620
G76	4	0.5714	1.0980
G77	3	0.4737	0.8011
G80	2	0.0000	0.1849
G81	4	0.6818	0.9351
G83	2	0.0000	0.2712
G87	3	0.1111	0.4258
G101	3	0.1500	0.3141
G102	2	0.0000	0.3768
G110	2	0.5500	0.6819
G116	3	0.0000	0.4634
G119	2	0.0526	0.2762
G120	2	0.0556	0.1269
G123	3	0.0435	0.2090
G128	2	0.2500	0.6919
G131	3	0.2609	0.4776
G133	3	0.6190	0.7920
G136	2	0.6667	0.6365
G142	2	0.6000	0.6730
G143	2	0.6667	0.6365
G145	2	0.6154	0.6172
G147	3	0.3571	0.7401
G150	2	0.4286	0.5196
G151	2	0.3000	0.4227
G154	4	0.5000	1.0251
G157	2	0.3889	0.6792
G165	2	0.8095	0.6749
G171	3	0.0000	0.4634
G174	2	0.1667	0.4029
G184	2	0.0000	0.3622
G185	2	0.0417	0.1013
G188	3	0.0667	0.5627
G191	2	0.2500	0.6616
G192	2	0.8667	0.6931
G200	4	0.9000	0.9386
G205	2	0.0000	0.6172
G206	2	0.0000	0.2868
G209	2	0.5000	0.5623
G211	2	0.0000	0.3768
G219	3	0.7500	0.9881
G225	2	0.4348	0.5236
G228	2	0.1176	0.3622
G233	3	0.2000	0.5627
G234	2	0.1250	0.4826
G244	3	0.9167	0.9222
G245	2	0.3500	0.4637
G249	2	0.5294	0.5779
G254	3	0.0909	0.3558
G262	2	0.0000	0.4506
G268	2	0.0000	0.1732
G269	2	0.0000	0.6365
G273	2	0.0000	0.1732
G284	2	0.0000	0.2237
G285	2	0.1739	0.2954

^1^The number of observed alleles.

^2^Estimated proportion of observed heterozygosity under random mating using Nei’s (1978) unbiased heterozygosity.

^3^Shannon's Information index (Lewontin, 1972).

### Genetic diversity study

**Figure 4:**
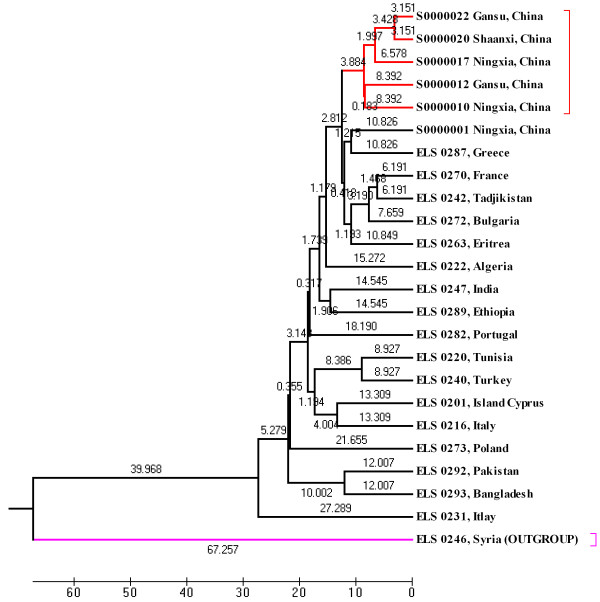
UPGMA dendrogram of 24 germplasm resources.

To assess the efficiency of microsatellites for differentiation of *L. sativus* from other *Lathyrus* species, we chose one *L. cicera* accession (ELS 0246, Syria) as outgroup in the genetic diversity study. Cluster analysis based on Nei’s [25] genetic distance indicated good separation between *L. sativus* and *L. cicera*. Furthermore, the UPGMA procedure grouped most Chinese accessions into one cluster; come from the center of origin, Mediterranean accessions discovered the major genetic diversity in cultivated grasspea species as they spread allover, except Chinese cluster (Figure 4). These results absolutely validated the accuracy and effectiveness of our approach for developing SSR markers in grasspea with the NGS technology.

## Discussion

### Grasspea as a potential vital crop in arid areas

Frequent drought and water shortage are worldwide problems, especially for agricultural production. Dryland agriculture plays an important role in national economy and food security. For example, in China, 55% of the total arable land, and 43% of the total food supplies are related to dryland agriculture. Grasspea is popular among the resource poor farmers in marginal areas due to the ease with which it can be grown successfully under adverse agro-climatic conditions without much production inputs. Presently at global scale, it is grown on 1.5 million ha area with 1.2 million tonnes production [2]. In recent years, efforts are underway in many countries including China, Australia, Spain, Italy, and Canada to expand its cultivation as a break crop between cereals and as a bonus crop in fallow land because of its ability to fix large amount of atmospheric nitrogen in association with *Rhizobium* bacteria [7]. However, the presence of a neurotoxin, β-N-Oxalyl-L-α,β-diaminopropionic acid (β-ODAP), renders this crop neglected and underutilized. Despite the undesirable features such as high neurotoxin, grasspea has potential as an important crop in western China and other arid areas in the world.

### Mining genomic SSR loci using 454 pyrosequencing technology

The traditional methods of microsatellite development used a library-based approach for targeted SSR repeat motifs, which was time consuming, expensive, with low-throughput. Hunting *in silico* for EST-SSRs from public database method is an alternative way, which was cost effective and easy to access. However, the total number of ESTs from grasspea and related species was very limited since grasspea has received less attention for molecular studies.

The identification of SSRs from genomic DNA using the 454 pyrosequencing technology was relatively new and two strategies were published. These were shotgun sequencing [16-18] and SSR-enriched sequencing [15,19]. In the present study, we used SSR-enriched sequencing technology and generated 370,079 high quality grasspea genomic reads, with an average length of 453 bp. Theoretically, the longer reads would increase our chances of successfully designing primer pairs while making it possible to identify long SSR repeats comparable to the size obtained using traditional library-based approach [18,27]. According to the MISA analysis, 651,827 SSRs were identified from 129,886 reads. This was a very positive result, as the high ratio of SSR-containing reads and the large number of putative SSRs we obtained. Among them, di- and tri-nucleotide repeat motifs dominated the grasspea genomic sequences, similar to findings in other crops [28]. (AC/GT)n was not only the predominant di-nucleotide repeat motif, but also the most frequent motif in the entire genome, accounting for 55.2% of the total SSRs, followed by (AAC/GTT)n, (AG/CT)n, (ACG/CTG)n, while, (AT/TA)n, (CG/GC)n, (CCG/CGG)n were rarely detected in this study. The pattern was moderately similar to that previously observed in faba bean [15]. Furthermore, isolated and identified low proportion of unwanted repeat motifs such as (AT/TA)n, (CG/GC)n, (CCG/CGG)n would enhance the success ratio in designing primers.

### Utilization of new SSR resources for ‘orphan crop’ grasspea research

Conventional breeding and phenotype research achieved great progress in improving agricultural crops in the last few years. However, grasspea was left as ‘orphan crop’ due to the lack of available genetic and genomic resources [29]. The use of SSR markers as a conventional tool has played an important role in the study of genetic diversity, genetic linkage map, QTL mapping and association mapping, and paved the way to the integration of genomics for crop breeding.

Due to the scarcity of user-friendly, highly polymorphic molecular markers in grasspea and other *Lathyrus* species, high-density genetic maps were not available. In the present study, we validated 288 non-redundant SSR primer pairs and 144 (50.0%) SSR primer pairs produced amplified bands, with 74 being polymorphic, and 70 monomorphic. This very large set of potential genomic-SSR markers will facilitate the construction of high-resolution maps for positional cloning and QTL mapping.

The genus *Lathyrus* L. (Fabaceae) is consisted of about 160 species [30] distributed throughout the temperate regions of the northern hemisphere and extends into tropical East Africa and South America [31,32]. This study, we used 74 new SSR primer pairs to clearly separate the 23 *L. sativus* accessions from one *L. cicera* accession, which is in agreement with the reported phylogenic studies of *Lathyrus* L. (Fabaceae) based on morphological and molecular markers [7,31].

## Conclusion

This study provides an extensive characterization of the SSRs in grasspea genome. For the first time, large-scale SSR-enriched sequence data was generated for the identification of SSRs and development of SSR markers to accelerate basic and applied genomics research in grasspea.

## Competing interests

The authors declare that they have no competing interests.

## Authors’ contributions

TY performed bioinformatic analysis, primer design and drafted the manuscript. JYJ created the SSR enriched DNA library and tested SSR markers. MB provided *L. sativus* accessions. JGH, CJC, SKA and RR assisted in designing experiment and preparing the manuscript. XLS and FW participated in 454 sequencing. JWC and XPH participated in quality inspection of the DNA library. JPG prepeared all the seed of *L. sativus*. XXZ designed and coordinated the study, and assisted in preparing the manuscript. All authors read and approved the final manuscript.

## Supplementary Material

Additional file 1: Table S1 The *Lathyrus sativus* L.and *Lathyrus cicera* L. germplasm used in this study. Click here for file

Additional file 2: Figure S1 Mononucleotide repeat motifs distribution. Click here for file

Additional file 3: Figure S2 Dinucleotide repeat motifs distribution. Click here for file

Additional file 4: Figure S3 Trinucleotide repeat motifs distribution. Click here for file

Additional file 5: Figure S4 Tetranucleotide repeat motifs distribution. Click here for file

Additional file 6: Figure S5 Pentanucleotide repeat motifs distribution. Click here for file

Additional file 7: Figure S6 Hexanucleotide repeat motifs distribution. Click here for file

Additional file 8: Table S2 All primers designed in this paper. Click here for file

## References

[B1] Campbell CG Mehra RB Agrawal SK Chen YZ Abd El Moneim AM Khawaja HIT Yadov CR Tay JU Araya WA Current status and future strategy in breeding grasspea (*Lathyrus sativus*) Euphytica 1993 73 1 167 175

[B2] Kumar S Bejiga G Ahmed S Nakkoul H Sarker A Genetic improvement of grass pea for low neurotoxin (β-ODAP) content Food Chem Toxicol 2011 49 3 589 600 10.1016/j.fct.2010.06.051 20659523

[B3] Patto M Skiba B Pang E Ochatt S Lambein F Rubiales D *Lathyrus* improvement for resistance against biotic and abiotic stresses: from classical breeding to marker assisted selection Euphytica 2006 147 1 133 147

[B4] Enneking D The nutritive value of grasspea (*Lathyrus sativus*) and allied species, their toxicity to animals and the role of malnutrition in neurolathyrism Food Chem Toxicol 2011 49 3 694 709 10.1016/j.fct.2010.11.029 21112364

[B5] Rybinski W Mutagenesis as a tool for improvement of traits in grasspea (*Lathyrus sativus* L.) Lathyrus Lathyrism Newsletter 2003 3 27 31

[B6] Yan Z-Y Spencer PS Li Z-X Liang Y-M Wang Y-F Wang C-Y Li F-M *Lathyrus sativus* (grass pea) and its neurotoxin ODAP Phytochemistry 2006 67 2 107 121 10.1016/j.phytochem.2005.10.022 16332380

[B7] Lioi L Sparvoli F Sonnante G Laghetti G Lupo F Zaccardelli M Characterization of Italian grasspea (*Lathyrus sativus* L.) germplasm using agronomic traits, biochemical and molecular markers Genet Resour Crop Evol 2011 58 3 425 437 10.1007/s10722-010-9589-x

[B8] Ponnaiah M Shiferaw E Pe ME Porceddu E Development and application of EST-SSRs for diversity analysis in Ethiopian grass pea Plant Genetic Resources 2011 9 2 276 280 10.1017/S1479262111000426

[B9] Sun X-L Yang T Guan J-P Ma Y Jiang J-Y Cao R Burlyaeva M Vishnyakova M Semenova E Bulyntsev S Zong X-X Development of 161 novel EST-SSR markers from *Lathyrus sativus* (Fabaceae) Am J Bot 2012 99 10 e379 e390 10.3732/ajb.1100346 23028003

[B10] Lucia L Incoronata G Development of genomic simple sequence repeat markers from an enriched genomic library of grass pea (*Lathyrus sativus* L) Plant Breed 2013 132 649 653 10.1111/pbr.12093

[B11] Mardis E The impact of next-generation sequencing technology on genetics Trends Genet 2008 24 133 141 10.1016/j.tig.2007.12.007 18262675 PMC2680276

[B12] Schuster SC Next-generation sequencing transforms today's biology Nat Meth 2008 5 1 16 18 10.1038/nmeth1156 18165802

[B13] Van Verk MC Hickman R Pieterse CMJ Van Wees SCM RNA-Seq: revelation of the messengers Trends in plant science 2013 18 4 175 179 10.1016/j.tplants.2013.02.001 23481128

[B14] Wang Z Gerstein M Snyder M RNA-Seq: a revolutionary tool for transcriptomics Nat Rev Genet 2009 10 1 57 63 10.1038/nrg2484 19015660 PMC2949280

[B15] Yang T Bao S Ford R Jia T Guan J He Y Sun X Jiang J Hao J Zhang X Zong X High-throughput novel microsatellite marker of faba bean via next generation sequencing BMC Genomics 2012 13 1 602 10.1186/1471-2164-13-602 23137291 PMC3542174

[B16] Tangphatsornruang S Somta P Uthaipaisanwong P Chanprasert J Sangsrakru D Seehalak W Sommanas W Tragoonrung S Srinives P Characterization of microsatellites and gene contents from genome shotgun sequences of mungbean (*Vigna radiata* (L.) Wilczek) BMC Plant Biology 2009 9 1 137 10.1186/1471-2229-9-137 19930676 PMC2788553

[B17] Csencsics D Brodbeck S Holderegger R Cost-Effective, Species-Specific Microsatellite Development for the Endangered Dwarf Bulrush (*Typha minima*) Using Next-Generation Sequencing Technology J Hered 2010 101 6 789 793 10.1093/jhered/esq069 20562212

[B18] Zhu H Senalik D McCown BH Zeldin EL Speers J Hyman J Bassil N Hummer K Simon PW Zalapa JE Mining and validation of pyrosequenced simple sequence repeats (SSRs) from American cranberry (*Vaccinium macrocarpon* Ait.) Theor Appl Genet 2012 124 1 87 96 10.1007/s00122-011-1689-2 21904845

[B19] Malausa T Gilles A Meglécz E Blanquart H Duthoy S Costedoat C Dubut V Pech N Castagnone-Sereno P Délye C Feau N Frey P Gauthier P Guillemaud T Hazard L Le Corre V Lung-Escarmant B Malé PJ Ferreira S Martin JF High-throughput microsatellite isolation through 454 GS-FLX Titanium pyrosequencing of enriched DNA libraries Mol Ecol Resour 2011 11 4 638 644 10.1111/j.1755-0998.2011.02992.x 21676194

[B20] Rice P Longden I Bleasby A EMBOSS: the European molecular biology open software suite Trends Genet 2000 16 276 277 10.1016/S0168-9525(00)02024-2 10827456

[B21] Thiel T Michalek W Varshney R Graner A Exploiting EST databases for the development and characterization of gene derived SSR markers in barley (*Hordeum vulgare* L.) Theor Appl Genet 2003 106 411 422 12589540 10.1007/s00122-002-1031-0

[B22] R Core Team R: A language and environment for statistical computing. R Foundation for Statistical Computing, Vienna, Austria 2012 ISBN 3-900051-07-0, URL http://www.R-project.org/

[B23] Rozen S Skaletsky H Primer3 on the www for general users and for biologist programmers Methods Mol Biol 2000 132 365 386 10547847 10.1385/1-59259-192-2:365

[B24] Yeh F Boyle T Population genetic analysis of co-dominant and dominant markers and quantitative traits Belgian Journal of Botany 1997 129 157

[B25] Nei M Estimation of average heterozygosity and genetic distance from a small number of individuals Genetics 1978 89 3 583 590 17248844 10.1093/genetics/89.3.583 PMC1213855

[B26] Tamura K Dudley J Nei M Kumar S MEGA4: molecular evolutionary genetics analysis (MEGA) software version 4.0 Mol Biol Evol 2007 24 1596 1599 10.1093/molbev/msm092 17488738

[B27] Zalapa JE Brunet J Guries RP Isolation and characterization of microsatellite markers for red elm (*Ulmus rubra* Muhl.) and cross-species amplification with Siberian elm (*Ulmus pumila* L.) Molecular Ecology Resources 2008 8 1 109 112 10.1111/j.1471-8286.2007.01805.x 21585729

[B28] Shi J Huang S Fu D Yu J Wang X Hua W Liu S Liu G Wang H Evolutionary dynamics of microsatellite distribution in plants: insight from the comparison of sequenced *Brassica, Arabidopsis * and other Angiosperm. PLoS One 2013 8 3 e59988 10.1371/journal.pone.0059988 23555856 PMC3610691

[B29] Varshney RK Close TJ Singh NK Hoisington DA Cook DR Orphan legume crops enter the genomics era! Current Opinion in Plant Biology 2009 12 2 202 210 10.1016/j.pbi.2008.12.004 19157958

[B30] Asmussen C Liston A Chloroplast DNA characters, phylogeny, and classification of *Lathyrus* (Fabaceae) Am J Bot 1998 85 3 387 10.2307/2446332 21684923

[B31] Leht M Phylogeny of Old World *Lathyrus* L. (Fabaceae) based on morphological data Feddes Repertorium 2009 120 1–2 59 74

[B32] Kupicha FK The infrageneric structure of *Lathyrus* Notes - Royal Botanic Garden Edinburgh 1983 41 2 209 244

