# Supplementary figures and images for: Large-scale microsatellite development in grasspea (Lathyrus sativus L.), an orphan legume of the arid areas
Source: BMC Plant Biol. 2014 Mar 17;14:65. doi: 10.1186/1471-2229-14-65 (PMC4003855; doi:10.1186/1471-2229-14-65)

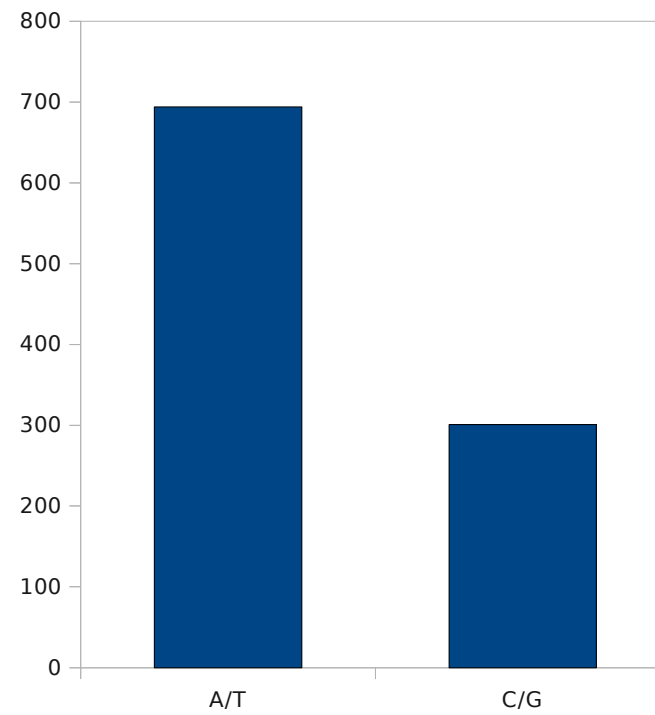

Figure S1 Mononucleotide repeat motifs distribution

Supplement: Additional file 2: Figure S1 — Mononucleotide repeat motifs distribution. [file 1471-2229-14-65-S2.pdf]

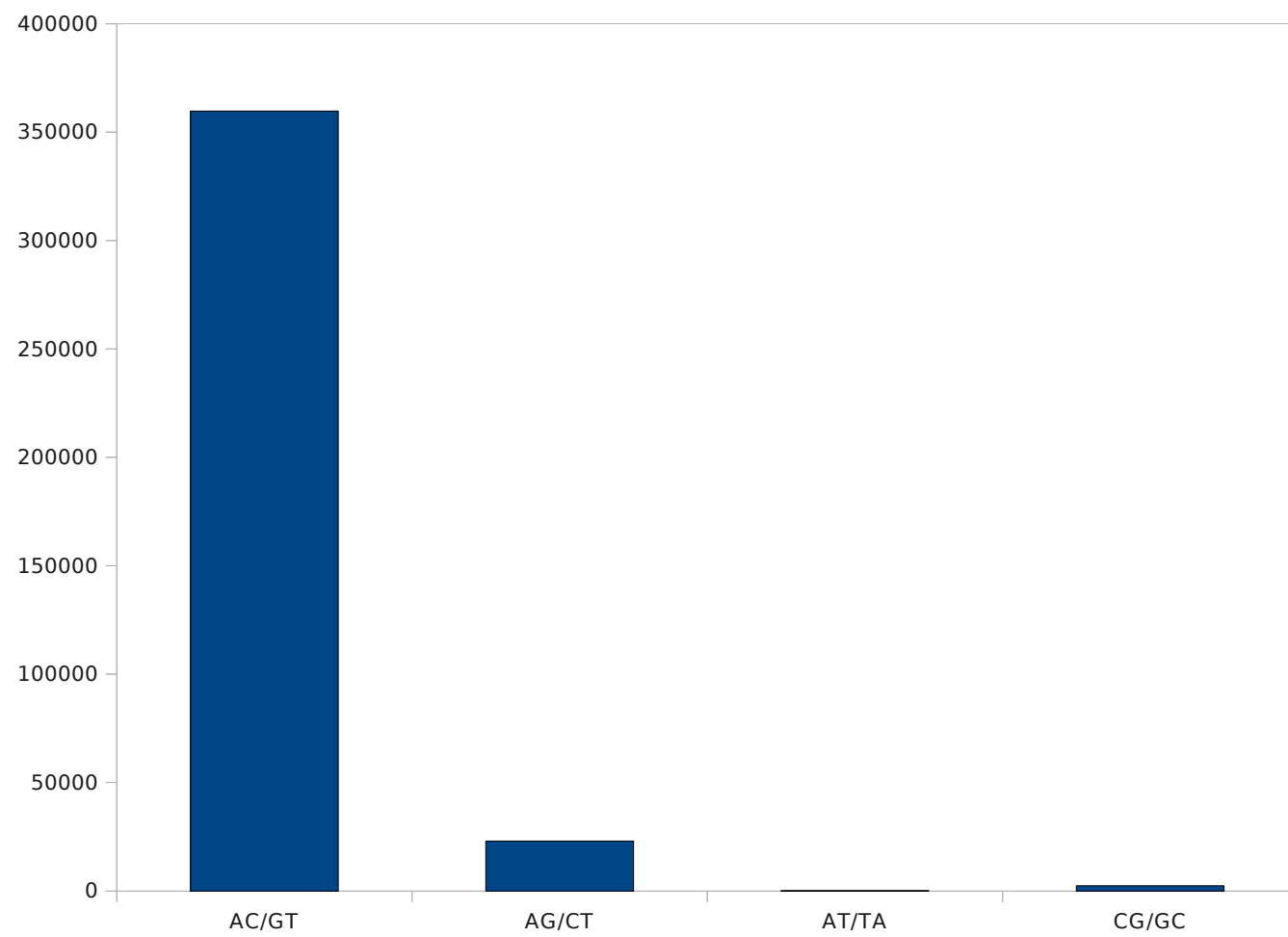

Figure S2 Dinucleotide repeat motifs distribution

Supplement: Additional file 3: Figure S2 — Dinucleotide repeat motifs distribution. [file 1471-2229-14-65-S3.pdf]

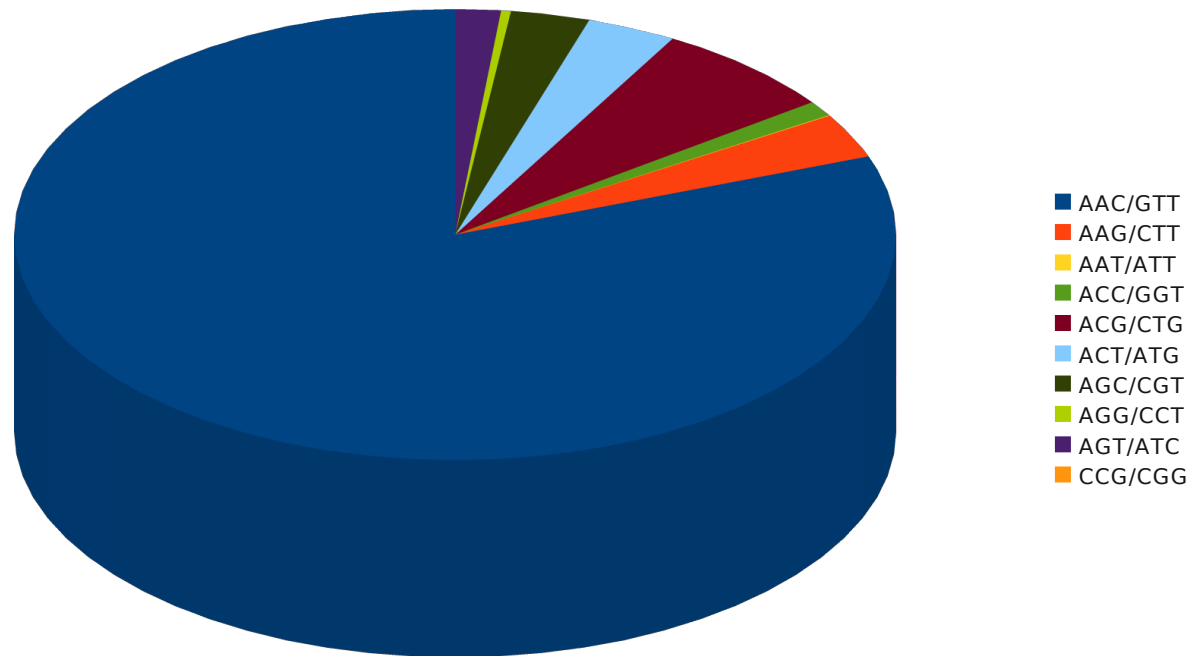

Figure S3 Trinucleotide repeat motifs distribution

Supplement: Additional file 4: Figure S3 — Trinucleotide repeat motifs distribution. [file 1471-2229-14-65-S4.pdf]

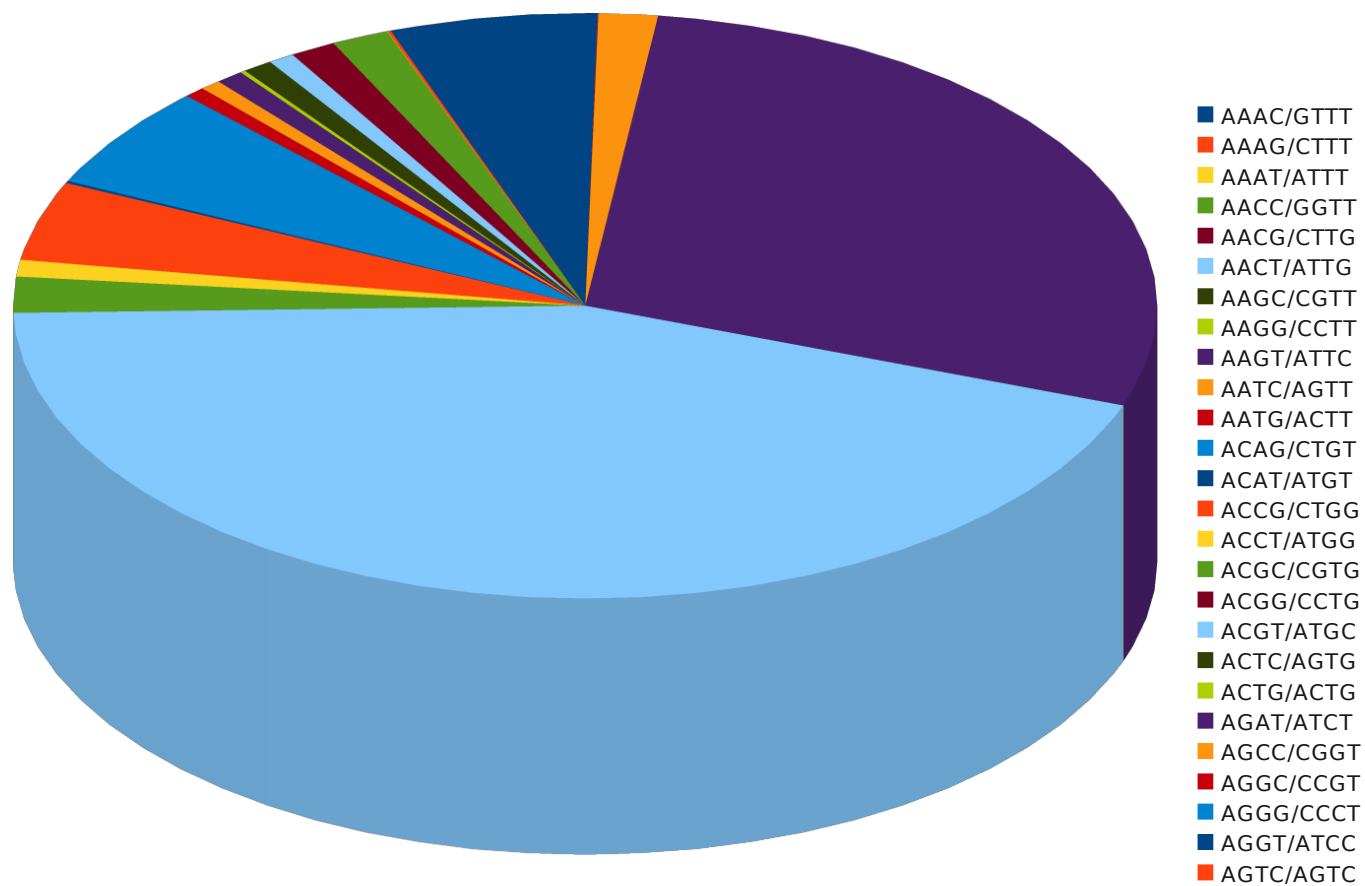

Figure S4 Tetranucleotide repeat motifs distribution

Supplement: Additional file 5: Figure S4 — Tetranucleotide repeat motifs distribution. [file 1471-2229-14-65-S5.pdf]

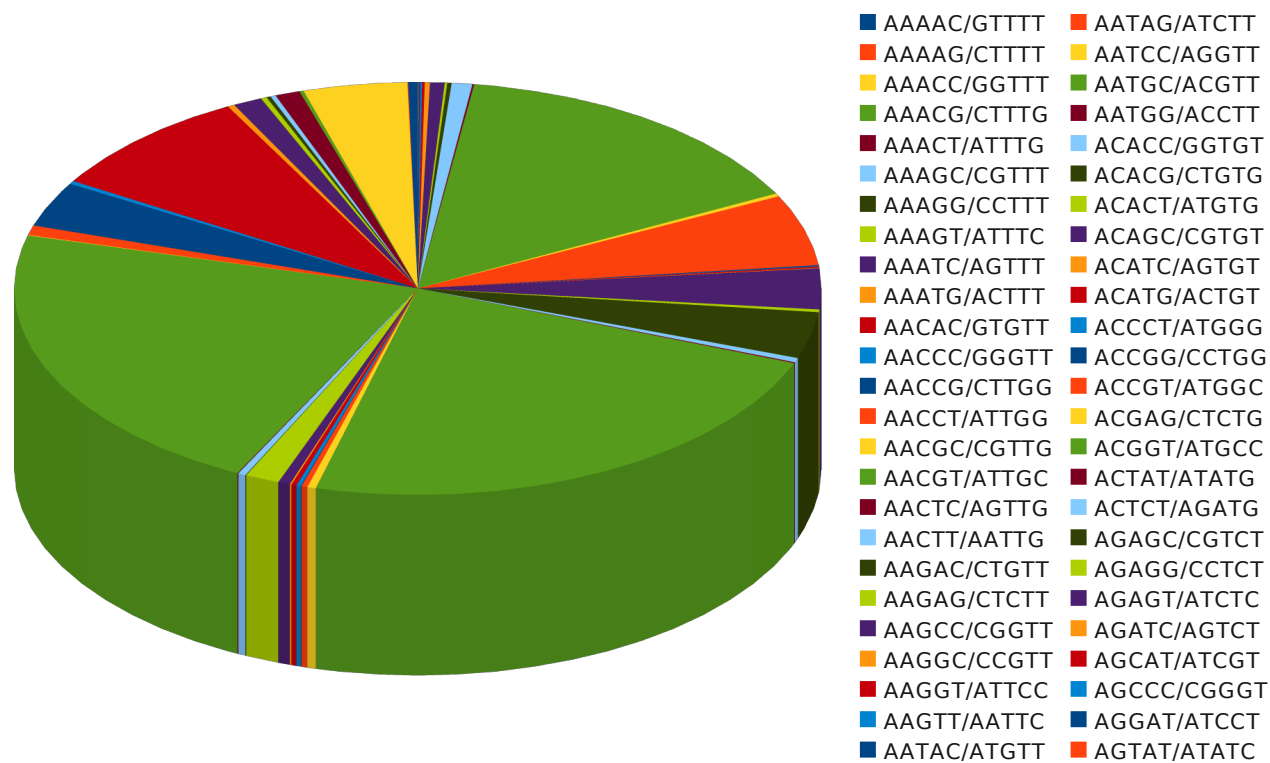

Figure S5 Pentanucleotide repeat motifs distribution

Supplement: Additional file 6: Figure S5 — Pentanucleotide repeat motifs distribution. [file 1471-2229-14-65-S6.pdf]

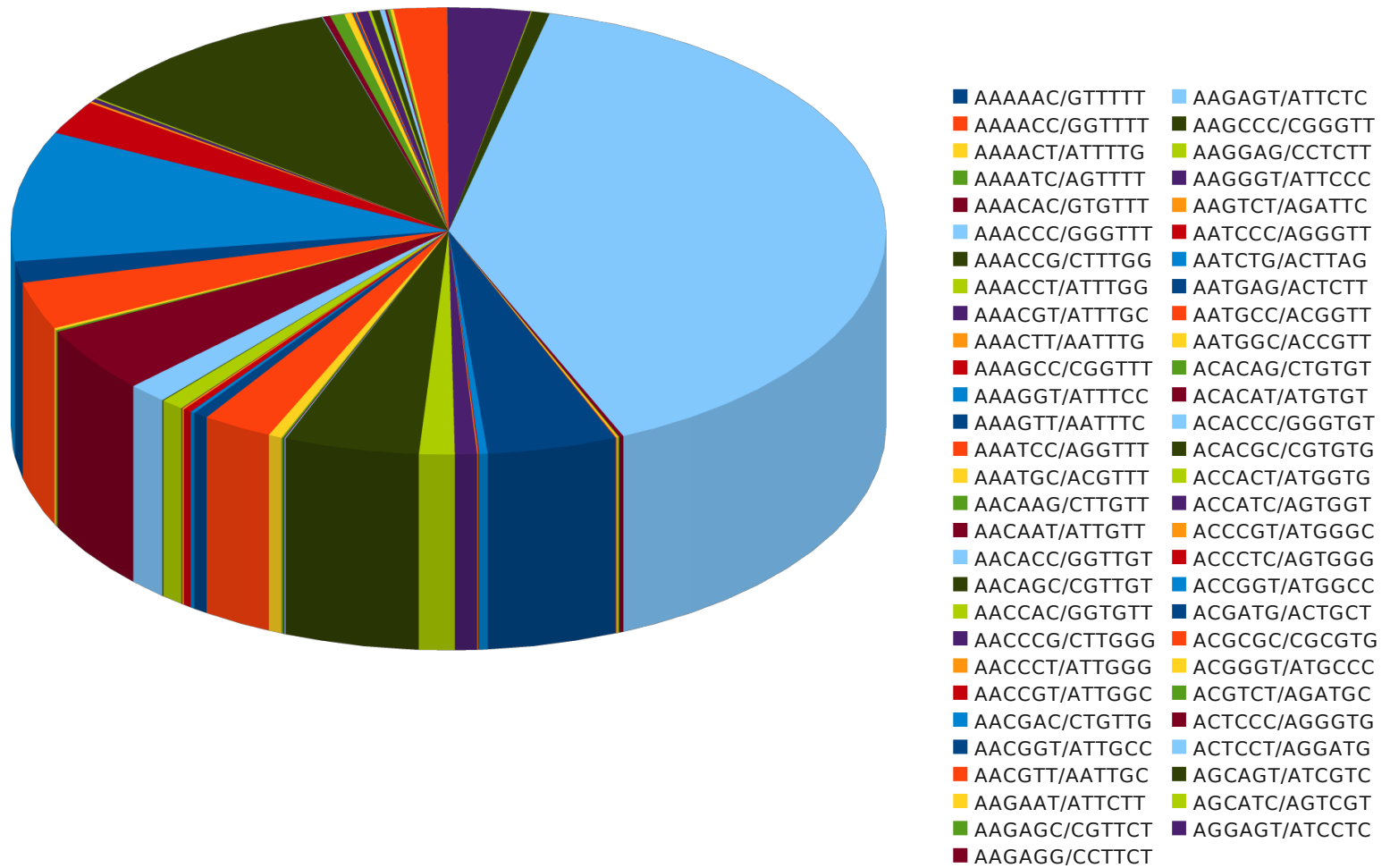

Figure S6 Hexanucleotide repeat motifs distribution

Supplement: Additional file 7: Figure S6 — Hexanucleotide repeat motifs distribution. [file 1471-2229-14-65-S7.pdf]
